# Notifiable condition reporting practices: implications for public health agency participation in a health information exchange

**DOI:** 10.1186/s12889-017-4156-4

**Published:** 2017-03-11

**Authors:** Debra Revere, Rebecca H. Hills, Brian E. Dixon, P. Joseph Gibson, Shaun J. Grannis

**Affiliations:** 1 School of Public Health, University of Washington, 1107 NE 45th St., Suite 400, PO Box 354809, Seattle, WA 98105 USA; 2 University of Washington, Seattle, WA USA; 3 Indiana University, Indianapolis, IN USA; 4 Marion County Public Health Department, Indianapolis, IN USA; 5 Regenstrief Institute, Indianapolis, IN USA

**Keywords:** Communicable Diseases, Disease Notification, Interprofessional Relations, Public Health Surveillance, Qualitative Research, Quality Control

## Abstract

**Background:**

The future of notifiable condition reporting in the United States is undergoing a transformation with the increasing development of Health Information Exchanges which support electronic data-sharing and -transfer networks and the wider adoption of electronic laboratory reporting. Communicable disease report forms originating in clinics are an important source of surveillance data for public health agencies. However, problems of poor data quality and delayed submission of reports to public health agencies are common. In addition, studies of barriers and facilitators to reporting have assumed that the primary reporter is the treating physician, although the extent to which a provider is involved in the reporting workflow is unclear. We sought to better understand the barriers to and burden of notifiable condition reporting from the perspectives of the three primary groups involved in reporting workflow: providers, clinic staff who bear the principal responsibility for reporting, and the public health workers who receive and process reports from clinics. In addition, we sought to situate these findings within the context of the future of notifiable disease reporting and the potential impacts of electronic lab and medical records on the surveillance system.

**Methods:**

Seven ambulatory care clinics and 3 public health agencies that are part of a Health Information Exchange in the state of Indiana, USA, participated in the study. Data were obtained from a survey of clinic physicians (*N* = 29), interviews with clinic reporters (*N* = 11), and interviews with public health workers (*N* = 9). Survey data were summarized descriptively and interview transcripts underwent qualitative analysis.

**Results:**

In both clinics and public health agencies, the laboratory report initiates reporting workflow. Provider involvement with reporting primarily revolves around ordering medications to treat a condition confirmed by the lab result. In clinics, reporting is typically the responsibility of clinic reporters who vary in frequency of reporting. We found an association between frequency of reporting, reporting knowledge and perceptions of reporting burden. In both clinics and public health agencies, interruptions and delays in reporting workflow are encountered due to inaccurate or missing information and impact reporting timeliness, data quality and report completeness. Both providers and clinic reporters lack clarity regarding how data submitted by their reports are used by public health agencies. It is possible that the value of reporting may be diminished when those responsible do not perceive receiving benefit in return. This may account for the low awareness of or recollection of public health communications with clinics that we observed. Despite the high likelihood that public health advisories and guidance are based, in part, on data submitted by clinics, a direct concordance may not be recognized.

**Conclusions:**

Unlike most studies of notifiable condition reporting, this study included the clinic reporters who bear primary responsibility for completing and submitting reports to public health agencies. A primary barrier to this reporting is timely and easy access to data. It is possible that expanded adoption of electronic health record and laboratory reporting systems will improve access to this data and reduce reporting the burden. However, a complete reliance on automatic electronic extraction of data requires caution and necessitates continued interfacing with clinic reporters for the foreseeable future—particularly for notifiable conditions that are high-impact, uncommon, prone to false positive readings by labs, or are hard to verify. An important finding of this study is the association between frequency of reporting, reporting knowledge and perceptions of reporting burden. Increased automation could result in even lower reporting knowledge and familiarity with reporting requirements which could actually increase reporters’ perception of notifiable condition reporting as burdensome. Another finding was of uncertainty regarding how data sent to public health agencies is used or provides clinical benefit. A strong recommendation generated by these findings is that, given their central role in reporting, clinic reporters are a significant target audience for public health outreach and education that aims to alleviate perceived reporting burden and improve reporting knowledge. In particular, communicating the benefits of public health’s use of the data may reduce a perceived lack of information reciprocity between clinical and public health organizations.

**Electronic supplementary material:**

The online version of this article (doi:10.1186/s12889-017-4156-4) contains supplementary material, which is available to authorized users.

## Background

In the United States, public health agencies (PHAs) collect reports about communicable disease cases to identify trends that merit public health response, and to decrease the opportunity for spread by assuring that patients are treated, that their contacts are tested, and that both are educated about the disease. Depending on state regulations regarding reporting, health care organizations or clinical laboratories (or both, in “dual reporting” states) are legally mandated to report cases of notifiable conditions to PHAs [1]. Data from these reports are consolidated, analyzed and summarized by PHAs to estimate the patterns and spread of disease in the community; assess effectiveness of control and prevention measures; identify high risk populations; formulate prevention strategies; allocate resources; and develop policies [2]. Clinical organizations benefit from the resulting information about disease occurrence and distribution, changes in risk factors and disease characteristics, and improved treatment recommendations or guidelines.

However, this information exchange suffers from the poor quality data captured on communicable disease report (CDR) forms; reports are incomplete and/or delayed and data are input into the wrong fields or erroneous [3–5]. In addition, reporting timeliness varies by disease, reporting protocol, surveillance goals and type of surveillance system. Timeliness can be improved with increased coordination between the clinical health care system and local/state PHAs [1]. The completeness of data reported for each case may be improved by increasing cross-jurisdictional coordination; implementing automated, electronic laboratory-based reporting; increasing the use of laboratory diagnostic tests in identifying new cases; and strengthening ties with those who are mandated to report communicable and infectious diseases [6].

Within the context of these recommendations, the most frequently investigated improvements involve leveraging electronic data collected from electronic laboratory reporting (ELR) systems and health information exchanges (HIEs). ELR systems can increase the identification of notifiable conditions [7], and improve completeness and timeliness of reporting to PHAs [8, 9]. This helps PHAs respond more rapidly to outbreaks, implement disease control measures, and monitor new and reemerging health threats. Similarly, HIEs, which can be conceived of as data sharing networks that facilitate electronic transmission of information among a group of health care organizations [10–12], also support improvements in reporting [13]. However, this potential is predicated on the assumption that electronic health records (EHRs) are available to PHAs through HIEs. Improved transmission of these medical data can support PHAs in more efficient investigation, contact tracing, case management and resolution and cluster identification [14, 15]. PHAs may also use HIEs to distribute information about outbreaks, emerging conditions and their associated treatment guidelines, and public health updates, alerts and advisories directly to the point-of-care to ensure providers take timely action in treating patients [16].

However, these promising improvements have limitations. Standard laboratory test result reports do not include detailed patient demographic information, treatment information, and other data PHAs need for effective surveillance. In addition, labs do not scrutinize test results to rule out false positives before they are sent to PHAs, which can increase investigation burden [17]. Regarding HIE data, combining information from multiple providers and different EHR systems requires extensive infrastructure support, technical skills, and workforce training. PHAs have very limited funding and resources for these tasks [15]. Also, EHR systems are structured to support the needs and workflow of clinical practice, not PH use and priorities. PHA use of EHR data transmitted by various EHR systems without an appropriate understanding of clinic workflow can result in reduced efficiency as PH workers process data submitted by clinic reporting systems that do not interoperate with one another or with PHA systems [16, 18].

This last point is critical when considering the barriers to reporting. One of the primary data sources for PHA disease surveillance is the CDR form submitted from a clinical setting. Several studies have investigated physicians’ perceived barriers to communicable disease reporting, finding that providers fail to report due to lack of awareness of their legal mandate to report, confusion regarding which specific diseases are reportable, lack of understanding how to report, need to protect patient privacy, and insufficient penalties for not reporting [5, 6, 19–22]. Despite this, there is little evidence to indicate that physicians bear the full responsibility for completing CDR forms as part of their clinic workflow nor that they are the principal agents for facilitating the flow of information between clinical and PHA settings, despite legal requirements to report [9, 23].

An HIE has the potential for leveraging EHR information from clinical settings along with electronic data collected through ELR systems to address the limitations of both in supplying complete and timely communicable and infectious disease data to PHAs. In this paper, we describe a study of CDR form processing in both clinic and PHA settings with an eye to better understanding how challenges to timely and complete notifiable condition reporting might be reduced by leveraging participation in a HIE.

## Objective

We report the results of an evaluation of notifiable condition reporting practices in ambulatory care clinics and CDR form processing in PHAs that are part of a statewide HIE. We sought to answer the following research questions:

What are the perceived barriers and facilitators to completeness of CDR forms and timely reporting of notifiable conditions to PHAs?

How do perspectives on these barriers and facilitators differ among providers, those at the clinics responsible for reporting and PHA staff responsible for processing CDR forms?

## Methods

### Settings and site selection

Our recruitment strategy sought diversity among ambulatory care clinics regarding provider and staff size, clinical specialty, number of patients and geographic location. Recruitment activities included outreach and informational sessions conducted by the research team. Clinic eligibility included being part of the Indiana Network for Patient Care HIE, willingness to complete the survey (by at least one provider/clinic) and willingness to participate in semi-structured interviews (by at least one person in the clinic whose responsibilities include CDR form completion and submission to PHAs, a type of targeted, purposive sampling). Recruitment outreach to nine ambulatory care clinics was conducted and seven agreed to participate.

PHA recruitment was based on clinic participation. The six enrolled urban clinics submit CDR forms to a larger health department which organizes reportable disease surveillance among 2 PHAs into two distinct working groups: sexually transmitted infections (STIs) and non-STIs. The rural clinic submits CDR forms to one rural PHA, which handles all reportable disease cases. Following clinic enrollment in the study, the corresponding PHAs (*N* = 3) were recruited into the study.

### Instrument development

Two instruments were developed for administration at the clinics. The *Provider Survey* collected information regarding reporting knowledge, practices and workflow; information exchanges between clinic reporters and PHAs; and perceptions of burden around, barriers to, and concerns about the reporting process. The survey included 80 items and took approximately 15 min to complete. See *Additional file* 1, *Provider Survey*, for details. A semi-structured *Clinic Reporter Interview Guide* covered content areas similar to the Provider Survey—reporting knowledge, practices and workflow; information exchanges between clinic reporters and PHAs; and perceptions of burden around, barriers to, and concerns about the reporting process. The Interview Guide contained 16 questions and interviews lasted 30–45 min. See *Additional file* 2, *Clinic Interview Guide*, for details.

One instrument, a semi-structured *Public Health Interview Guide* was designed to capture PHA practitioners’ experiences and workflow with CDR forms; data quality, report completeness and timeliness issues with reporting; communications with clinics and labs around reporting; and perceived barriers to reporting and information exchange. The Interview Guide contained 17 questions and interviews lasted approximately 40 min. See *Additional file* 3, *Public Health Interview Guide*, for details.

Pilot testing of the survey and interview guides was conducted internally to streamline questions, remove redundancies, and calculate timing.

### Data collection

Data were collected between September 2012 and April 2014. Participation in all study activities was voluntary and administrative methods differed depending on protocol, as follows. Paper copies of the provider survey were distributed to and picked up from the enrolled clinics in September 2012. Participation in the survey was anonymous: no identifiers of individual subjects, specific clinics or association of any individual participation with a specific clinic was made. Twenty-nine surveys were returned.

Two researchers experienced in qualitative and mixed-methods (DR, RH) conducted all telephone and in-person interviews. Following oral informed consent to participate, clinic reporter interviews (*n* = 11) were conducted at urban clinics between September 2012 and January 2013 and at the rural clinic in July 2013. An on-site project liaison at each clinic identified potential interviewees based on their notifiable condition reporting responsibilities within the clinic. Depending on responsibilities, additional clinic reporters were recruited using snowball sampling. Following oral consent to participate, public health interviews (*n* = 9) were conducted in person at the urban PHAs in September 2013 and by phone at the rural PHA in April 2014. Managers at each PHA identified individuals for interview who were primarily responsible for CDR form receipt, processing and case investigation. All interviews were audio-recorded.

### Data analysis

#### Surveys

Data from surveys (*N* = 29) were extracted into Microsoft Excel [24]. Descriptive statistics were used to characterize the sample and their responses; open-ended responses were categorized. Histograms were created to display the frequency for which each rating scale category was utilized. After examination of frequencies it was determined that, per standard Rasch rating scale optimization [25], the five-point Likert survey scales could be reduced to three categories: Never/Seldom, Sometimes, and Often/Always.

#### Interviews

Interviews (*N* = 20) were transcribed verbatim by a professional transcriptionist to yield 227 pages of transcription for import into the NVivo qualitative data analysis (QDA) software [26]. Two researchers (RH, DR) reviewed each interview transcript in full to identify key coding concepts and themes based on the study research questions and to develop an initial open coding scheme using the framework of grounded theory [27]. Researchers then independently coded two randomly selected transcripts (one clinic, one PHA) using the initial coding scheme, identified new codes, and uncovered problems and discrepancies which were resolved by consensus [27, 28]. This process continued through three iterations (6 clinic, 4 PHA transcripts; 50% of the transcripts). Redundant codes were collapsed, and more general codes were split until the coders reached agreement on a final codebook. Both researchers then divided up the remaining transcripts for coding and periodically met to discuss results. A final check of coding was conducted on 2 randomly selected transcripts (1 clinic, 1 PHA) to ensure inter-coder reliability. QDA software was assistive in generating coding summaries and creating code co-occurrences or associations, which were used to inform thematic development. Codes were collated into themes to qualitatively summarize knowledge of notifiable condition reporting rules; frequency of reporting; reporting workflow within clinic and PHA settings; protocols for handling missing information; information exchanges between clinic and PHA settings; and barriers to reporting as perceived by clinic reporters and PHA notifiable condition investigators.

#### Synthesis

Metadata regarding clinic reporter- and PHA-specific codes was maintained throughout the coding process to facilitate interpretation of results. Associations between codes and their meta-data/context (site; setting; respondent characteristics) were determined and code and code co-occurrence frequencies were calculated. Themes were developed by one researcher (DR) using a mixed methods enumeration process [29, 30] to generate interpretations, identify relationships among themes, and integrate these interpretations with quantitative results.

## Results

Due to differences in administration, survey and interview results are reported separately.

### Population characteristics

Characteristics of the enrolled clinics are summarized in Table 1. There were a total of 228 providers practicing across the 7 clinic locations. Among the providers, 215 (94.3%) were medical doctors (MDs) while 11 (4.8%) were nurse practitioners. Four sites provide primary care regardless of age or gender, while one site specializes in primary care for young women, especially sexually active women; one clinic specializes in primary care for individuals 18 years and older; and one clinic specializes in primary care for women. All but one clinic is located in an urban, metropolitan setting. Five of the clinic use electronic lab orders, and all but one clinic faxes communicable disease reports to the local PHA.

**Table 1:** Enrolled clinic characteristics

Clinic	Location	Provider Type: Number	Service	# patients/month	Mode: Lab Orders	Mode: CDR → PHA
1	Urban	MD:9; NP:4	Primary Care	1000	electronic	fax
2	Urban	MD:140; NP:5	Primary Care	6700	electronic	fax out of EMR
3	Urban	MD:8	Teen Clinic	1000	electronic	fax
4	Urban	MD:37; NP:1; PA:2	Adult Medicine	2860	electronic	fax/mail
5	Urban	MD:10; NP:1	Primary Care	2600	electronic	mail
6	Urban	MD:9	Women’s Health	1000	paper, fax	fax
7	Rural	MD:2	Primary Care	1200	paper	fax

*MD* Medical Doctor, *NP* Nurse Practitioner, *PA* Physician’s Assistant, *CDR* Communicable Disease Reporting, *PHA* Public Health Agency

### Provider surveys

Surveys provide a contextual description of provider notifiable condition reporting knowledge, workflow, experience and perception of burden. While we are unable to report clinic-specific response rates due to the anonymity protocol, 29 surveys were returned for 12.7% overall response rate. The majority of respondents were female (79%) physicians (79%) working in primary care (86%). Level of respondent experience varied but approximately one-third (35%) had been in practice for less than five years.

### Knowledge and experience with reporting

Table 2 summarizes provider knowledge of and experience with notifiable condition reporting: 86% of providers reported that their clinic or organization had specific protocols for reporting; 60.7% reported having ever completed a CDR form; and few providers indicated they received training on notifiable conditions from a PHA in the last year (21%), received a list of reportable conditions (28%), or were familiar with the variability in state-mandated reporting time frames for different conditions (17%).

**Table 2:** Provider knowledge of & experience with notifiable condition reporting & forms

	Yes N (%)
Have you ever completed a notifiable condition report form?	17 (61)
In the past year, have you received any training about Indiana’s reportable conditions requirements?	6 (21)
Does your clinic or organization have specific protocols for reporting cases to public health or the health department?	24 (86)
In the past year, were you provided with a list of conditions to report to public health or the health department?	8 (28)
Are you familiar with the different time frames for reporting specific notifiable conditions to public health or the health department?	5 (17)
In the past year, did you receive any calls from public health or the health department regarding a reportable case?	3 (11)
In the past year, did you need to call public health or the health department regarding a reportable case?	8 (29)

### Barriers to reporting

**Fig. 1:**
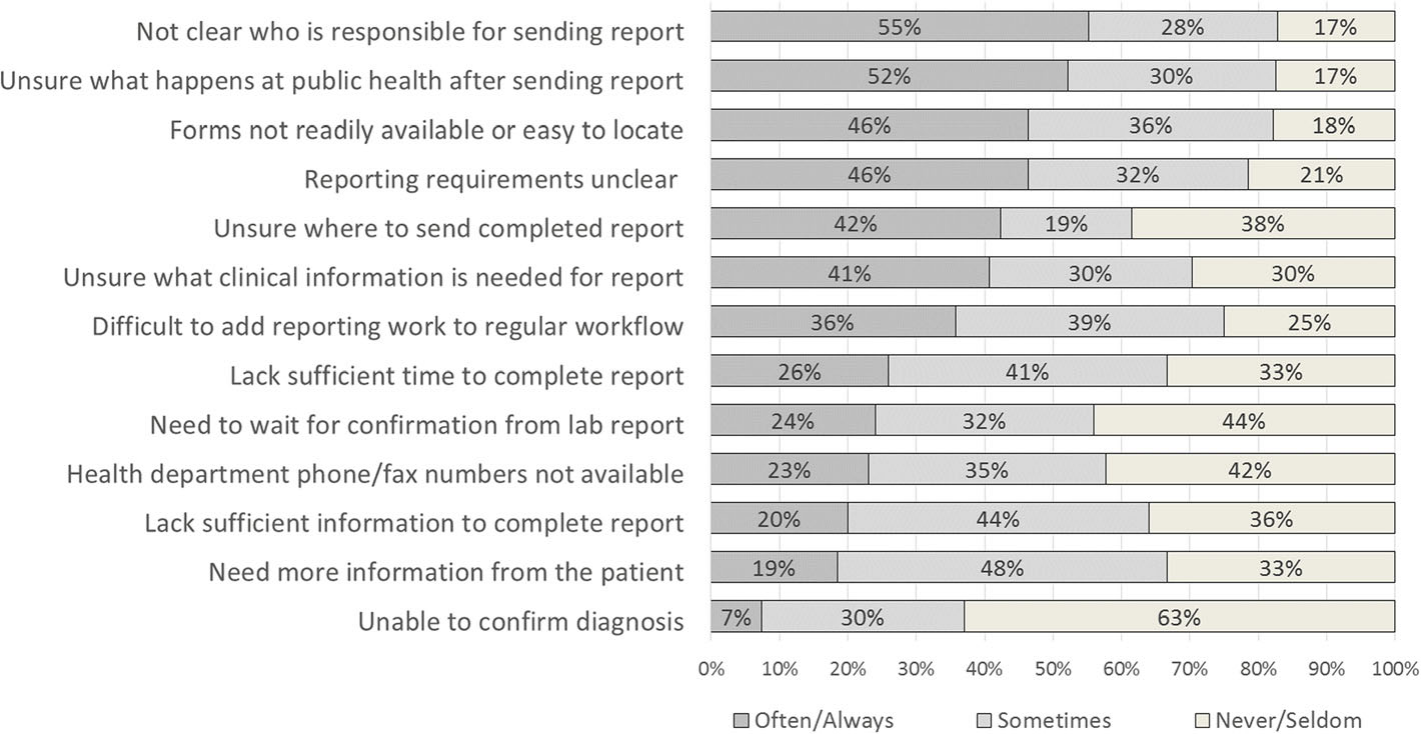
Provider Survey: Frequency at which providers encounter specific barriers to reporting

When asked to rate the frequency with which they encounter various known barriers or problems related to notifiable condition reporting, providers most frequently reported uncertainty about who was responsible for reporting (55%), uncertainty about how reports were used by PHAs (52%), lack of clarity about reporting requirements (46%), and difficulty locating CDR forms at the clinic (46%). Other issues often encountered included uncertainty regarding where to send a completed CDR form (42.3%), what clinical information is needed for a report (41%), and difficulties incorporating reporting into regular workflow (36%). In open-ended items, providers also reported confusion about how and what to report as well as concerns about the time needed to complete a report. Figure 1 summarizes providers’ perceived barriers to reporting.

### Clinic reporter and PHA staff interviews

#### Overview

Clinic reporter and PHA staff interviews, respectively, captured in-depth information regarding the experiences of those responsible for completing CDR forms and those processing CDR form information. Five major themes emerged from qualitative analysis of the interviews, each with a number of sub-themes as described in Table 3.

**Table 3:** Major themes with frequencies of sub-themes coded in clinic reporter (*n* = 11) and PHA worker (*n* = 9) interviews

		Clinics n (%)	PHAs n (%)
*Theme 1: Greater reporting knowledge and experience is associated with perceptions of a minimal reporting burden*			
*Sub-themes:*	Notifiable condition reporting is not a burden or time-consuming activity or interruptive of daily workflow for regular reporters	7 (63.6)	-
	Reporting requirements (who should report, which conditions are reportable, which forms to use, reporting timeframes for different conditions) are clear for regular reporters	7 (63.6)	-
	Clinic settings in which regular reporters work have a well-established flow of information and process for handling new positive cases of reportable conditions	7 (63.6)	-
	Infrequent reporters perceive notifiable condition reporting as burdensome and interruptive of their workflow	4 (36.4)	-
	Reporting requirements are not clear for infrequent reporters	4 (36.4)	-
	Infrequent reporters assume that labs report notifiable conditions	4 (36.4)	-
	Infrequent reporters express confusion about whether their organization requires notifiable condition reporting	4 (36.4)	-
*Theme 2: A positive laboratory report initiates the case reporting process in both clinic and PHA settings*			
*Sub-themes:*	Reporting workflow in begins with receipt of a positive lab result in both clinics and PHAs	11 (100)	9 (100)
	PHA workers begin case processing activities with receipt of a positive lab result	-	9 (100)
	Other than treatment orders based on a lab report, physicians are not involved reporting workflow	11 (100)	
	Lab reports are missing critical information, such as clinic name, patient phone number, etc., so are insufficient alone for case reporting	-	9 (100)
	Delays in lab reporting contribute to delayed CDR form completion	3 (27.3)	
*Theme 3: Inaccurate or missing information interrupts and delays reporting which contributes to timeliness*, *data quality and completeness issues*			
Sub-themes:	PHA workers perceive the majority of CDR forms they receive as generally incomplete, missing crucial information and low in data quality	-	9 (100)
	PHA workers report frequent communications with clinics to gather needed case information	-	9 (100)
	PHA workers perceive that communications with clinics around reporting can be unproductive and frustrating	-	9 (100)
	Clinics are perceived as infrequent reporters due to their assumption that labs report to PHAs	-	9 (100)
	Specific to communications around notifiable condition reporting, clinic reporters perceive frequency of contact from PHAs as rare	8 (72.7)	-
	Inaccurate or missing contact information prevents reaching patients regarding treatment	6 (54.5)	9 (100)
	Clinic reporters assume labs report so do not regularly submit CDR forms	4 (36.4)	-
	Clinic reporters are unaware that they are required to submit CDR forms	4 (36.4)	-
	Clinic reporters knowingly submit CDR forms with missing information	3 (27.3)	-
	Clinic reporters only complete CDR form fields that they deem pertinent	2 (18.2)	-
*Theme 4: Searching for needed information interrupts and delays reporting and case investigation workflow*			
Sub-themes:	Numerous and varied information resources are utilized to complete CDR forms, conduct investigations and/or close cases	9 (81.8)	9 (100)
	Clinic reporters spend time looking for, waiting for and compiling information from various sources (EHRs, different reporting and/or clinical systems, chart notes, lab reports, online searches, etc.)	7 (63.6)	-
	PHA workers spend a majority of their time looking for and compiling information from various sources to conduct case processing	-	9 (100)
*Theme 5: PHAs cannot be certain that the clinical advisories*, *updates and information they send are reaching their target audience*			
Sub-themes:	Clinic reporters do not have a clear idea about how information such as CDR form data is used by PHAs	8 (72.7)	-
	Announcements and information sent by PHAs (fax, email) are not routinely distributed throughout the clinic	5 (45.5)	-
	Some clinics report they never receive announcements or information from PHAs	4 (36.4)	-
	Only PHA information deemed relevant is disseminated but how that determination is made is unclear to recipients	3 (27.3)	-

While physicians are involved in ordering treatment for a notifiable condition, at most clinics the MAs, RNs or LPNs are responsible for completing and submitting CDR forms. Among those interviewed, clinic reporters described variability in frequency of reporting and proportion of frequently reported conditions, with STI conditions such as Chlamydia cited as most frequently reported. Overall, clinic reporters spend a small proportion of their work time on reporting, although we identified a distinction between those we characterized as *regular reporters* (7/11 interviewed; 63.6%) from those who *infrequently report* (4/11 interviewed; 36.4%). For *regular reporters*, reporting was described as a simple, straightforward task, requiring little time or effort, and the clinic settings in which they worked had a well-established flow of information and process for handling new positive cases of reportable conditions. In contrast, *infrequent reporters* perceived notifiable condition reporting as a burdensome, unclear and interruptive task and expressed confusion about who should report, which conditions are reportable, which forms to use, the reporting timeframes for different conditions, and whether their organization even required notifiable condition reporting.

For PHA workers, the bulk of their workday is spent handling reports and the burden associated with notifiable condition reporting revolved around CDR forms themselves, which PHA workers perceived as generally incomplete, missing crucial information and low in data quality. Low reporting volume by clinics was perceived as the norm by the PHA workers—a norm PHA workers attributed to a misperception on the part of clinics that lab reporting alone is sufficient. PHA workers were so accustomed to poor reporting from clinics that they routinely begin the case investigation process when they receive the laboratory report, rather than waiting for the clinic-submitted CDR form, even though it could contain needed information such as treatment details, patient demographics and contact information, or confirmation of the diagnosis by a physician.

In the following section we focus on those qualitative themes most germane to the context of increased opportunities for leveraging available EHR information from clinical settings and expanded ELR systems within a HIE.

#### Theme: A positive laboratory report initiates the case reporting process in both clinic and PHA settings

The lab report is a central actor in the reporting process. The trigger for reporting in the clinic, as well as beginning a case report in the PHA, is receipt of a positive lab result. At the clinic, when completion of a CDR form is initiated by the positive lab result, the workflow can be described as a process of repeatedly duplicating information across systems.

*“We have the [CDR] form that we fax, we put that form in the person’s medical record chart, put information in the computer, put our notes in there, and then they’re printing [the CDR form] and putting it in the chart. So there’s a lot of double-duty.” [Clinic 3, Respondent 1]*

As stated earlier, PHA workers routinely begin the case investigation process when they receive the laboratory report, rather than waiting for the clinic-submitted CDR form which could contain needed information such as treatment details, patient demographics, and contact information.

*“It’s rare to get a complete lab report. But typically when I get things from a lab they don’t have all of the history or anything like that…” [PHA 3, Respondent 1]*

“*Sometimes we only receive the patient’s name and test result on the* [*lab] report. And then the doctor’s office will say they gave all the information to the lab and wonder why we’re bothering them.”* [*PHA 1, Respondent 1]*

Initiating case processing with the lab report can contribute to duplication of effort and additional processing for PHA workers as a lab result for one case may be delivered from the processing lab, the state reporting system, and clinic CDR reports—all at different times.

“*When we get the labs, whether it’s fax or in the mail or electronically, it’s all there so when we get it, we just look at it to see who it’s from, print off whatever information, and start our investigation. And then the lab might come through, it might not, so we already begin—so we have duplicates with the lab coming in and the provider but we*, *sometimes we already call the provider before we get it and we might have to go back and ask them to get the information. Or I just look into the computer to get it*, *because we have to look everyone up anyway*.[..*] It’s just a lot of paper work that we’re getting*, *duplicates.”* [*PHA 1*, *Respondent 3]*

#### Theme: Inaccurate or missing information interrupts and delays reporting contributing to timeliness, data quality and completeness issues

As noted above, lab reports generally trigger case investigations. However, several challenges to producing CDR forms that contain timely, complete and accurate information were identified. In particular, patient demographics and information may be either missing or inaccurate on those reports. For example, when a patient checks in for her appointment at the clinic, she may change her phone number on a paper registration form but the EHR may not be updated for several days. When a lab for this patient is ordered at the appointment it may have the patient’s old phone number on it, if it contains any contact information at all. The lab test that is returned then and, if positive, simultaneously sent to the PHA will then contain this old contact information.

For some conditions, such as Chlamydia, CDR forms require documenting the date the patient began treatment. In these cases, the provider must order treatment or the patient must visit the clinic to receive treatment which causes further delay.

“*It might take you a month to get a hold of some of our patients.”* [*Clinic 2*, *Respondent 2]*

Some clinic reporters assumed that reporting by the labs was sufficient while other confirmed that they intentionally submit incomplete or erroneous reports when they deem the missing information is unnecessary.

“*I know those conditions are getting reported and it must be by the lab.”* [*Clinic 4*, *Respondent 1]*

“*I think that the vast majority of our providers and nurses*, *you know*, *think that the lab takes care of all of that.”* [*Clinic 5*, *Respondent 1]*

“*We don’t fill out all of* [*the form]. We fill out the things that are pertinent.”* [*Clinic 3*, *Respondent 1]*

“*…some patients*, *we can’t get a hold of so we go ahead and fax* [*the incomplete CDR form] to the health department and let them manage it.”* [*Clinic 2*, *Respondent 1]*

This is consistent with the perspective of PHA workers, that CDR forms from clinics cannot be relied on.

“[*reporters] leave out a lot of fields*, *whether it’s their treatment*, *or symptoms*, *or titer.”* [*PHA 2*, *Respondent 4]*

“*It’s unusual … to have detailed info on a report…”* [*PHA 1*, *Respondent 2]*

#### Theme: Searching for needed information interrupts and delays reporting and case investigation workflow

Both clinic reporters and PHA workers need to utilize numerous and varied information resources to complete CDR forms, conduct investigations and close cases. To complete CDR forms, clinic reporters spend time looking for, waiting for and compiling information from various sources—EHRs, different reporting and/or clinical systems, chart notes, lab reports, online searches, etc. When information is missing from regular sources, clinic reporters will search for the needed information which can delay submitting completed CDR forms to the PHA.

“[*We look] in the chart…the EMR*, *but because the notes aren’t always in there*, *we may have to cobble that together. And all the demographics come from a different system.”* [*Clinic 1*, *Respondent 1]*

“*A lot of* [*our] patients don’t have up-to-date contact numbers*, *so we may have to call their emergency contact or the pharmacy and ask if they’ve got an updated phone number for them.”* [*Clinic 6*, *Respondent 1]*

Similarly, PHA workers utilize numerous and varied information resources to conduct investigations and/or close cases. Even when they receive clinic CDR forms, these are generally perceived as incomplete, missing crucial information and low in data quality which necessitates searching for information from a variety of resources.

“*We look in InSight* [*local PHA database of labs*, *CDRs and case investigations]*, *CareWeb* [*Web-based viewer of integrated EHRs]…the medical records section…we use Facebook*, *peoplesearch.com*, *411*, *any kind of resource… Google the person’s name and a lot of the time we can find information on them.”* [*PHA 2*, *Respondent 4]*

“*And we look up* [*Department of Corrections] records. Because the state forms ask us*, *was the person incarcerated?”* [*PHA 1*, *Respondent 3]*

“*We have a rolodex to find the doctor’s information. Google—Google is my best friend if I don’t have something*, *I will Google immediately so that’s the big thing that I use. Or I use Healthgrades and WebMD*. [..*] sometimes the number on the lab won’t match*, *or we’ll get the lab electronically and there’ll be the name of the provider but not the phone number or there’ll be the name of the provider and the address but no phone number so I’ll do a Google search on the address or on the doctor to get the phone number so that I can call for information.”* [*PHA 2*, *Respondent 3]*

PHA workers described frequent contact with provider offices, hospitals, and labs when searching for missing, essential CDR form information. Yet clinic reporters perceive frequency of contact from PHAs as rare.

“*I spend approximately two hours on my phone every day* [*to clinics for CDR form information].”* [*PHA 1*, *Respondent 3]*

“*The phone calls back and forth are typically the biggest* [*delay].”* [*PHA 1*, *Respondent 5]*

“*I’ve never had anyone from the health department call me and say that they have questions*, *or unable to get ahold of the patient*, *or anything.”* [*Clinic 6*, *Respondent 1]*

### Synthesis

#### CDR form completion is typically the responsibility of clinic reporters, not providers

Provider involvement with reporting primarily revolves around ordering medications to treat a condition confirmed by the lab result. Providers were unfamiliar with reporting workflow, reporting requirements or how to report. Providers overall report uncertainty regarding notifiable condition reporting rules, responsibilities, and protocols―which could be expected given their lower responsibility for reporting compared to other clinical team members. They are also perceived as less knowledgeable by both clinic reporters and PHA workers.

Principal responsibility for reporting rests on clinic reporters, who vary in frequency of reporting. We found an **association between frequency of reporting, reporting knowledge and perceptions of reporting burden**. Providers, who rarely report, are not familiar with the list of legally reportable conditions or the timeframes for reporting. We found that regular reporters had a more efficient reporting workflow, greater comfort and familiarity with reporting protocols, spent minimal time on reporting activities, and associated little burden with reporting. Infrequent clinic reporters found reporting more burdensome and time-consuming, an unwelcome diversion from regular workflow, and expressed a lack of clarity about processes for CDR form completion and submission to PHAs. However, we are unable to determine any directionality, i.e., whether better workflow leads to more reporting or vice versa.

While **a positive laboratory report initiates the case reporting process in both clinic and PHA settings**, for providers lab results primarily serve as a trigger to order treatment while clinic reporters are tasked to initiate the reporting process. In PHAs, workers often do not wait for CDR forms from clinics, but rather begin case investigation activities based on lab results they receive, regardless of limited information provided on lab reports. Both settings encounter **interruptions and delays in reporting workflow due to inaccurate or missing information**. Issues of reporting timeliness, data quality and completeness impact both clinic reporters and PHA workers who spend **significant time and effort searching for information**. Particularly for PHA workers, the overwhelming amount of time spent on information seeking could be significantly reduced if CDR forms were completed on time and contained accurate information.

Both providers surveyed (52%) and clinic reporters (72.7%) **lack clarity regarding how CDR form reports or their data are used by PHAs**. It is possible that the value and importance of reporting may be diminished when those responsible for reporting do not perceive receiving benefit from submitting notifiable condition data to PHAs or perceive a lack of information reciprocity with PHAs. This may account for the seemingly low awareness of or recollection of communications from PHAs or with PHA workers, as well as low levels of PHA information distribution within clinics. Despite the high likelihood that advisories and guidance disseminated by PHAs are based, in part, on data submitted by clinics, a direct concordance may not be recognized.

## Discussion

To better situate potential improvements to notifiable condition reporting within HIE advances and the expansion of ELR, we sought to understand the perceived barriers and facilitators to completeness of CDR forms and timely reporting of notifiable conditions to PHAs and how perspectives on these barriers and facilitators might differ among providers, those at the clinics responsible for reporting and the PHA staff responsible for processing CDR forms. Unlike most studies of notifiable condition reporting, this study did not focus solely on physicians or hospital settings,[5, 6, 19, 23, 31] but included the ambulatory care clinic reporters who bear the primary responsibility for completing CDR forms and are most actively involved in their clinic’s reporting workflow and tasks.

A primary barrier to clinic notifiable condition reporting is timely and easy access to data by clinic reporters without interruptions to workflow or normal clinic tasks. By collecting and sharing data across health care organizations, HIE networks are significantly transforming the work of both clinical and public health, with increased opportunity for more automated and efficient data capture [16]. It is possible that expanded adoption of HIE will improve access to data and automate its extraction. By making it easier to collect required data through electronic request from a single source, the burden of information gathering by both clinic reporters and PHA workers might be mitigated with consequent quality improvements [32].

We found that lab reports initiate the case reporting workflow in both clinics and PHAs. However, lab reports do not provide the data PHAs require to complete a case report, launch an investigation or close a case, such as demographic and other patient information, which are often missing or incorrect in lab reports. And although clinic reporters bear the burden for reporting it must be noted that, given a positive lab report is the trigger for case reporting, the role of the provider in ordering a lab test might be examined more closely. While we found variation in clinic reporter frequency it is possible that this variation is more an outcome of provider lab-request variation which might impact or possibly bias disease notification. The decision to order a laboratory request may reflect provider’s perception of disease threat, illness severity, utility of laboratory-based case definitions or, as in the case of a diagnosis that requires multiple testing or clinical symptoms, insufficient presenting evidence to warrant a test order [5].

In the US, health departments currently receive up to 62% of their total laboratory-based reports for notifiable diseases through ELRs [33]. And as noted earlier, ELR systems can increase the identification of notifiable conditions [7] and improve timeliness and completeness of reporting to PHAs [8, 9]. Expanded EHR adoption in conjunction with connectivity with an ELR system might further support automation of notifiable condition report data capture and its delivery to PHAs. Through participation in a HIE, PHAs could potentially benefit from the development of systems that can automatically pre-populate CDR forms with available electronic data from the patient’s health record and from the lab report. This approach is currently under investigation in a pilot project being conducted in a HIE by a team of researchers that includes the authors (see [34] for more information).

However, automation, either through EHR or ELR, is not a silver bullet. EHRs continue to have quality and completeness problems [35] so a complete reliance on automatic electronic extraction of data requires caution and necessitates continued interfacing with clinic reporters for the foreseeable future. In addition, despite improving quality and timeliness of data extraction by EHR and ELR systems, some notifiable conditions that are high-impact, uncommon, prone to false positive readings by labs, or are hard to verify will still require confirmation by providers and clinic reporters; otherwise, PHAs will continue to be burdened by CDR forms that may be missing information, contain inaccurate information, or should not have been delivered to PHAs at all—all of which can drain limited PHA investigation resources. Again, while there are emerging efforts to build infrastructure that enables greater automation of case reporting, these efforts are nascent and must be evaluated as to their impact on both clinical and PH workflows.

An important finding of this study is the association between frequency of reporting, reporting knowledge and perceptions of reporting burden. Increased automation through expanded EHR and ELR systems will further lower the human investment required for reporting. It is possible this could result in even lower reporting knowledge and familiarity with reporting requirements in the clinical setting, which could actually increase reporters’ perception of notifiable condition reporting as burdensome. In addition, we also found that there was uncertainty, even among regular clinic reporters, about how submitted CDR forms are used by PHAs. Information reciprocity between a PHA and its stakeholders is a standard component of surveillance [36]. A recent study reported that sharing results generated by information submitted by stakeholders such as clinic providers and reporters will demonstrate the utility and value of information sharing while ensuring PHA analyses of submitted information can be utilized by stakeholders in their own work [37].

Outcomes of this study point to several recommendations and next steps. Given their central role in reporting, clinic reporters are a significant target audience for PHA outreach and education that aims to alleviate perceived reporting burden and improve reporting knowledge. PHA training of clinic reporters around communicable and infectious disease reporting may be helpful, especially if focused on the PH process and importance of the information, such as specific CDR form fields, to PH surveillance. PHA outreach around surveillance and reporting may improve both provider and clinic reporter perceptions regarding the importance of timely and complete clinical data for measuring disease trends, effectively applying control and prevention measures, identifying high risk populations or geographic areas, and keeping the clinical community informed through alerts, advisories, updates, and guidelines. Reinforcing the value of data delivered by providers and clinic reporters to PHAs may improve sense of partnership and cement the commonalities shared between clinic and PHA organizations.

## Limitations

There are several limitations to this study. Because the surveys were completed anonymously, we were unable to associate survey results with specific clinics or their characteristics. Also, purposive sampling—in which we specifically requested to interview clinic staff who were tasked with notifiable condition reporting responsibilities and most familiar with CDR form completion—may not result in a representative sample so we are unable to state that the experiences and perceptions of those we interviewed can be generalized widely. However, given clinics varied in size, specialty area, and location, we believe our findings can be applied beyond the specific clinics or clinic reporters participating in the study.

## Conclusions

Previous studies investigating notifiable condition reporting have focused on providers instead of the RNs and MAs principally responsible for clinical reporting. These clinic reporters may be a more appropriate audiences for PHA outreach and education efforts targeted at improving reporting knowledge, frequency, timeliness and data quality. Specifically, disseminating information about how reported data are used and the importance of individual form fields for both public and clinical health surveillance could serve to address the perceived lack of information reciprocity and improve clinical reporting measures. The potential for EHR and ELR adoption and use to support electronic case reporting is limited today and impact on clinic and PHA workflow requires thoughtful attention.

## Additional files

Additional file 1: Provider Survey. (DOCX 28 kb)
Additional file 2: Clinic Reporter Interview Guide. (DOCX 19 kb)
Additional file 3: Public Health Worker Interview Guide. (DOCX 18 kb)

## Abbreviations

CDR form: Communicable disease report form
EHR: Electronic health record
ELR: Electronic laboratory reporting
HIE: Health information exchange
PH: Public health
PHA: Public health agency
QDA: Qualitative data analysis
STI: Sexually transmitted infection
