# Supplementary material for: Notifiable condition reporting practices: implications for public health agency participation in a health information exchange
Source: BMC Public Health. 2017 Mar 11;17:247. doi: 10.1186/s12889-017-4156-4 (PMC5346201; doi:10.1186/s12889-017-4156-4)
Supplement: Additional file 1: — Provider Survey. (DOCX 28 kb) [file 12889_2017_4156_MOESM1_ESM.docx]

**Provider Survey**

**SECTION 1: BACKGROUND**

| A. What is your health care provider specialty? | check one |
| --- | --- |
| Physician |  |
| Nurse |  |
| Pharmacist |  |
| Physician's Assistant |  |
| Medical Assistant |  |
| Administrator |  |
| Other (please fill in) | |

| B. What is your work setting? | check one |
| --- | --- |
| Primary Care |  |
| Emergency Care |  |
| Specialty Care |  |
| Public Health |  |
| Other (please fill in) | |

| C. How long have you been in your  present occupation? | check one |
| --- | --- |
| < 5 years |  |
| 5-10 years |  |
| 11-15 years |  |
| 16-20 years |  |
| > 20 years |  |

| D. What is your primary specialty? | check one |
| --- | --- |
|  |  |
| Emergency Medicine |  |
| Internal Medicine |  |
| Family Practice |  |
| Pediatrics |  |
| Obstetrics & Gynecology |  |
| Pharmacy |  |
| Specialty Practice |  |
| Other (please fill in) | |

| E. How long have you worked at your present workplace? | check one |
| --- | --- |
| < 5 years |  |
| 5-10 years |  |
| 11-15 years |  |
| > 15 years |  |

| F. What is your gender? | check one |
| --- | --- |
| Male |  |
| Female |  |

| G. What is your age? | check one |
| --- | --- |
| 21-29 years |  |
| 30-39 years |  |
| 40-49 years |  |
| 50-59 years |  |
| > 60 years |  |

| **SECTION 2: EXPERIENCE WITH NOTIFIABLE CONDITION REPORTING** | | |
| --- | --- | --- |
| 1. Have you received any training about Indiana's reportable conditions requirements in the past year? | yes | no |
| 2. Does your clinic or organization have specific protocols for reporting notifiable conditions to public health or the health department? | yes | no |
| 3. In the past year, were you provided with a list of conditions to report to public health or the health department? | yes | no |
| 4. Are you familiar with the different time frames for reporting specific notifiable conditions to public health or the health department? | yes | no |
| 5. In the past 6 months, did you receive any health alerts, advisories, or updates from public health or the health department regarding infectious or communicable diseases? | yes | no  SKIP TO Q 6 |
| 5A. How many alerts, advisories or updates would you estimate you received in the past 6 months? |  | |
| 6. In the past 6 months, did you receive any calls from public health or the health department regarding a reportable conditions form? | yes | no  SKIP TO Q 7 |
| 6A. How many calls would you estimate you received in the past 6 months? |  | |
| 7. In the past 6 months, did you need to call public health or the health department regarding a reportable conditions form? | yes | no  SKIP TO Q 8 |
| 7a. How many calls would you estimate you made in the past 6 months? |  | |
| 8. How long would you estimate it takes to complete a reportable conditions form? |  | |
| 9. How many people in your practice setting would you estimate are involved in completing a reportable conditions form? |  | |
| 10. Have you **ever** completed a reportable conditions form? | yes | no  SKIP to Q 11 |
| 10a. Please circle the type of form(s) you have completed | paper | electronic |

| **SECTION 3: BARRIERS TO REPORTING**  11. Several factors have been identified as barriers to reporting of notifiable conditions and completion of reporting forms. On a scale of 1 to 5, please rate how **frequently** you encounter the following barriers: | | | | | |
| --- | --- | --- | --- | --- | --- |
|  | 1  Never | 2  Seldom | 3  Some-times | 4  Often | 5  Always |
| a. Reporting requirements are unclear |  |  |  |  |  |
| b. Waiting for confirmation from a lab report |  |  |  |  |  |
| c. Unsure what clinical data is needed for a report |  |  |  |  |  |
| d. Unable to complete diagnosis |  |  |  |  |  |
| e. Need more information from the patient |  |  |  |  |  |
| f. Do not have sufficient information to complete a report |  |  |  |  |  |
| g. Not enough time |  |  |  |  |  |
| h. Health department phone/fax numbers are not available |  |  |  |  |  |
| 12. Are there other factors that you find are barriers to completing reportable conditions forms? | | | yes | no  SKIP TO Q 14 | |
| 13. Please list other barriers you have encountered regarding completing reportable conditions forms: | | | | | |
| 14. Overall, which of the factors listed above do you find to be the largest barrier to reporting notifiable conditions? | | | | | |
| 15. Do you have any other observations, thoughts or experiences regarding completion of reportable conditions forms or interactions with public health or the health department? | | | | | |

| **Thank you for completing this survey!** |
| --- |
