# Supplementary material for: Notifiable condition reporting practices: implications for public health agency participation in a health information exchange
Source: BMC Public Health. 2017 Mar 11;17:247. doi: 10.1186/s12889-017-4156-4 (PMC5346201; doi:10.1186/s12889-017-4156-4)
Supplement: Additional file 2: — Clinic Reporter Interview Guide. (DOCX 19 kb) [file 12889_2017_4156_MOESM2_ESM.docx]

**Clinic Reporter Interview Guide**

**1. Background:** I'd like to start with a little background information

1A. What is your title? (check full- or part-time) How long have you worked at [SITE]?

1B. Could you describe the kind of health care setting [SITE] is, kinds of patients seen, etc?

1C. What notifiable conditions are reported most frequently at [SITE]? (prompt with list if needed)

1D. How frequently would you say [SITE] sends a report to public health? (monthly?)

**2. Notifiable Conditions & Reporting:** We're interested in how reportable conditions are handled at [SITE].

2A. Let's say a patient is suspected of having [condition mentioned in 1C]. Could you walk me through the steps taken at [SITE] to handle this?

Prompts: Who is involved (check re: MD)? What is your role in this process? How is information communicated? Systems/tools used to gather information or complete forms? How are labs and lab paperwork are handled? Estimated time from start to end?

2B. Let's say [SITE] receives a lab report that a patient has tested positive for [condition mentioned in 1C]. Could you walk me though the steps that would be taken to handle reporting this case to public health?

Prompts: Who is involved (check re: MD)? What is your role in this process? How is information communicated? Systems/tools used to gather information or complete forms? How paperwork is handled? Where are CDR forms kept (Paper or electronic? Location? Ease of access)? How are they sent to PHA (method? Specific person?)? Is there a specific time of day or day of week that a report will be sent to PHA? After a form is sent is it destroyed or archived? Estimated time from start to end?

**3. Barriers & Burden:** As you know we're interested in learning about the kinds of issues that can impact reporting to public health or filling out a CDR form.

3A. Have you received any training or instruction on how to report to public health? (Probe: details).

3B. (Ask respondent to looking at blank CDR form): What fields do you usually complete before sending the report to PHA? Do some fields take more time to complete than others? Are some fields problematic?

3C. What do you do when you need to find information, like treatment confirmation or titer, to complete a form? What resources do you use to find this information (EHR, paper, databases, people)? If forms are held while waiting for information, where are they kept?

**4. Communications**: We'd like to learn a bit more about interactions you might have with PHAs.

4A. In the last 6 months, have you had to communicate with the health department about a patient with a communicable or infectious disease? (Prompt: Phone? FAX? Time spent?)

4B. In the last 6 months, have you received communications from the health department about case you reported? (Prompt: Phone? FAX? Time spent?)

4C. How does [SITE] receive health alerts, advisories, or updates from the health department? (Prompt: Phone? FAX? Listserv?) Is this information circulated among providers and staff at [SITE]? How is this done? (Prompt: Email, meetings, copied FAX, print-outs posted to bulletin boards)

**5. Close:** Is there anything else you'd like to share with us about notifiable condition reporting or the work you do around reporting, how public health information is handled at [SITE] or disease surveillance in general?

Thanks again for your time. Do you have any questions for us?
