# Supplementary material for: Notifiable condition reporting practices: implications for public health agency participation in a health information exchange
Source: BMC Public Health. 2017 Mar 11;17:247. doi: 10.1186/s12889-017-4156-4 (PMC5346201; doi:10.1186/s12889-017-4156-4)
Supplement: Additional file 3: — Public Health Worker Interview Guide. (DOCX 18 kb) [file 12889_2017_4156_MOESM3_ESM.docx]

**Public Health Worker Interview Guide**

**1. Background:** I'd like to start with a little background information

1A. What is your title? (check full- or part-time) How long have you worked at SITE?

1B. What is your role with respect to notifiable condition reporting?

1C. Do you focus on any specific notifiable conditions or populations (ex, hep B and pregnant women)?

**2. Case Processing:** We're interested in how you handle, process and close a typical case.

2A. Thinking of [condition mentioned in 1C], could you walk me through the steps you take, from first bit of information you receive to the the point at which you close the case?

**3. Time:** We're interested in the amount of time you spend handling reports **during a typical week**.

3A. How much time would you estimate you spend working with notifiable condition reports? How many reports were you working with during that time? (Probe: distribution of conditions)

3B. How much time would you estimate you spend gathering (or tracking down) information that wasn't included on CDR forms or lab reports or was unclear for the cases you were handling?

**4. Barriers & Burden**: We'd like to learn a bit more about how you gather and review information for closing a case.

4A. On a scale from 1 to 10 where 1 is "blank" and 10 is "complete", how complete would you say most reports are that you receive from outpatient clinics? Labs? Hospitals? Other?

4B. Typically, what information is missing? Inaccurate? Needs clarifying? (Probe: differences among reporters?)

4C. On a scale from 1 to 10 where 1 is "never arrives" and 10 is "right on time", how timely would you say most reports are that you receive from outpatient clinics? Labs? Hospitals? Other?

4D. How do you handle getting information because what you need is missing? Needs confirmation? Is delayed in getting to you?

Probe re method and amount of time: Phone? Fax? Letters? Databases? Internet? Etc.

Probe re source: Clinic (how do you know who to contact)? Lab? Patient? Other PHAs?

Probe re frequency? How often do you need to do this in a typical week?

4E. Are you able to access information you need electronically from the patient’s medical record? From other electronic sources (state surveillance system)?

4F. Some cases, like Chlamydia, need treatment confirmation. How long do you wait for information like this?

4G. Are there other ways you track down information?

4H. In your experience, do you find that some notifiable condition cases or conditions seem to take more time to close than others? (Probe: What might account for this?)

**5. Close:** Is there anything else about handling notifiable condition reports or closing cases that you would like to share?

Thank you again for your time. Do you have any questions for us?
